# Perceptions of ChatGPT in healthcare: usefulness, trust, and risk

**DOI:** 10.3389/fpubh.2024.1457131

**Published:** 2024-09-13

**Authors:** Su-Yen Chen, H. Y. Kuo, Shu-Hao Chang

**Affiliations:** ^1^Institute of Learning Sciences and Technologies, National Tsing Hua University, Hsinchu, Taiwan; ^2^Department of Sport Management, College of Health and Human Performance, University of Florida, Gainesville, FL, United States

**Keywords:** healthcare, clinical practice, medical education, medical research, technology acceptance model, ChatGPT, uses and gratifications theory, Taiwan

## Abstract

**Introduction:**

This study explores the perceptions of ChatGPT in healthcare settings in Taiwan, focusing on its usefulness, trust, and associated risks. As AI technologies like ChatGPT increasingly influence various sectors, their potential in public health education, promotion, medical education, and clinical practice is significant but not without challenges. The study aims to assess how individuals with and without healthcare-related education perceive and adopt ChatGPT, contributing to a deeper understanding of AI’s role in enhancing public health outcomes.

**Methods:**

An online survey was conducted among 659 university and graduate students, all of whom had prior experience using ChatGPT. The survey measured perceptions of ChatGPT’s ease of use, novelty, usefulness, trust, and risk, particularly within clinical practice, medical education, and research settings. Multiple linear regression models were used to analyze how these factors influence perception in healthcare applications, comparing responses between healthcare majors and non-healthcare majors.

**Results:**

The study revealed that both healthcare and non-healthcare majors find ChatGPT more useful in medical education and research than in clinical practice. Regression analysis revealed that for healthcare majors, general trust is crucial for ChatGPT’s adoption in clinical practice and influences its use in medical education and research. For non-healthcare majors, novelty, perceived general usefulness, and trust are key predictors. Interestingly, while healthcare majors were cautious about ease of use, fearing it might increase risk, non-healthcare majors associated increased complexity with greater trust.

**Conclusion:**

This study highlights the varying expectations between healthcare and non-healthcare majors regarding ChatGPT’s role in healthcare. The findings suggest the need for AI applications to be tailored to address specific user needs, particularly in clinical practice, where trust and reliability are paramount. Additionally, the potential of AI tools like ChatGPT to contribute to public health education and promotion is significant, as these technologies can enhance health literacy and encourage behavior change. These insights can inform future healthcare practices and policies by guiding the thoughtful and effective integration of AI tools like ChatGPT, ensuring they complement clinical judgment, enhance educational outcomes, support research integrity, and ultimately contribute to improved public health outcomes.

## Introduction

ChatGPT, developed by OpenAI, is a chatbot powered by a large language model (LLM) that utilizes deep learning and natural language processing (NLP) techniques. Since its launch on November 30, 2022, ChatGPT has rapidly gained widespread popularity, amassing over 100 million users within two months. This remarkable growth underscores the significant interest in and potential applications of ChatGPT across various sectors. Consequently, its ability to generate coherent and varied text responses, a key feature of generative AI, positions it as a valuable tool for healthcare, supporting clinical, educational, and research activities (1, 2). Understanding how different user groups, particularly those in healthcare and non-healthcare fields, perceive this technology is crucial for its effective adoption and integration into healthcare environments. This study aims to contribute to the strategic development of AI technologies and support broader public health objectives by enhancing the quality and accessibility of healthcare through technological advancements.

The potential of ChatGPT to reshape healthcare has been extensively explored in academic literature, highlighting its significant impact in various fields. Scholars have emphasized ChatGPT’s potential and challenges in clinical, teaching, and research applications, stressing the importance of addressing issues of accuracy, transparency, and ethics (1). This multifaceted impact suggests a significant future role for ChatGPT in enhancing medical education and patient care, provided it operates within a responsible and ethical framework (1–11).

In clinical applications, ChatGPT has shown promise in diagnosis and decision-making. Studies have explored ChatGPT’s capabilities in clinical diagnostics, highlighting both its potential and constraints in handling diverse clinical issues. Evaluations by major medical institutions suggest that while ChatGPT can enhance decision-making and efficiency, medical professionals must be aware of its capabilities and limitations, advocating for cautious use in clinical settings (5, 7). Additionally, another study illustrates how ChatGPT could be effectively integrated into medical consultations, particularly when used alongside other multiagent models, to enhance clinic practices (11).

Similarly, in medical education, ChatGPT has shown potential in developing curricula, enhancing teaching methodologies, creating personalized study plans, and improving teaching efficiency (3, 6). Scholars also note its impact on enhancing student learning and clinical reasoning skills. Research illustrates ChatGPT’s effectiveness in preparing students for exams like the USMLE and various Chinese medical licensing examinations, suggesting its potential for students’ self-directed leaning in test preparation, and for transforming educational and assessment materials (12, 13). However, these advancements also bring challenges such as potential for academic dishonesty, and the need for a multicultural approach in teaching methodologies.

In medical research, ChatGPT’s influence is growing as it assists with medical writing, literature summary, and research review (3). It can support the advancement of the academic community in medical research by reducing the burden of critical appraisal and research reporting, facilitating knowledge dissemination, and generating novel research idea for knowledge creation (1, 2). Despite these benefits, challenges related to misinformation, inconsistency, and the risk of overdependence on technology must be addressed through careful management and continuous oversight to fully leverage the benefits of AI in medical research while mitigating associated risks (2–4).

The discussion on key ethical principles for using AI in healthcare is essential, particularly in ensuring that AI tools like ChatGPT are aligned with human values and acknowledging that AI cannot replace the moral reasoning and critical reflection provided by human professionals (6). It is also vital to focus on fairness and the prevention of exacerbating existing biases in healthcare (1). The deployment of generative AI in healthcare must prioritize accountability and transparency in clinical decision-making (9), while also addressing the risks of misinformation (8).

AI has significant potential in clinical settings, especially in decision-making and workflow integration; however, notable limitations exist. While AI tools like ChatGPT demonstrate impressive accuracy, they require continuous information input and careful interpretation to avoid errors in differential diagnosis and clinical management (7). Additionally, concerns about the reliability and comprehensiveness of AI-generated responses, the inability to interpret images, and the challenges of maintaining up-to-date and accurate information underscore the need for further validation and transparency in AI applications (1, 2, 5). The use of AI in healthcare, particularly with tools like ChatGPT, presents significant risks, including the potential for generating inaccurate or biased information. These risks are heightened with free versions of AI models, which may lack necessary updates, real-time data access, and security measures, increasing the likelihood of misinformation.

Literature on ChatGPT usage behaviors predominantly employs two main theoretical frameworks: technology acceptance model (TAM) and Uses and Gratifications Theory. TAM is instrumental in investigating how users come to accept and utilize new technologies, focusing on factors such as perceived usefulness, ease of use, trust, and perceived risk. On the other hand, the Uses and Gratifications Theory explores how individuals engage with new technologies, with a particular focus on user experience, motivations, and gratifications derived from the technology.

Several studies have examined perceptions and acceptance of ChatGPT across diverse user groups, offering insights into the factors that influence its adoption. For example, a survey of healthcare and non-healthcare professionals in India revealed that healthcare professionals generally had a more positive outlook on ChatGPT, despite lower usage rates compared to non-healthcare professionals (14). In the Middle East, a modified TAM for ChatGPT identified that perceived risk and attitudes toward technology significantly influence user acceptance among healthcare students, with ease of use and positive attitudes being crucial for fostering favorable views (15, 16). Similarly, research involving university students in Oman found that while ChatGPT’s perceived usefulness drove adoption for educational purposes, ease of use was not a significant factor in their intentions (17).

The Uses and Gratifications Theory further elucidates user engagement with ChatGPT. A study from Norway identified productivity and novelty as key motivations within this framework, highlighting ChatGPT’s potential as a reliable educational and collaborative tool (18). Research on American users, which applied measurement items from existing Uses and Gratifications literature, found that personalization reduced perceived creepiness and increased trust, enhancing user confidence and intentions to continue using the technology (19). Additionally, a survey involving Chinese users adapted TAM into an AI Device Use Acceptance (AIDUA) model, incorporating Cognitive Appraisal Theory (CAT). This study revealed that social influence, novelty value, and perceived humanness positively influenced performance expectancy (perceived usefulness), while these factors, along with hedonic motivation, negatively impacted effort expectancy (ease of use) (20).

Furthermore, specific applications of ChatGPT in healthcare have been explored in recent studies, examining perceptions from both the general public and healthcare professionals. In the United States, a survey found a positive relationship between general trust in ChatGPT and both the intention to use and actual use of the AI, particularly for health-related queries (21). Another study highlighted a significant willingness among users to rely on ChatGPT for self-diagnosis, pointing to the need for safe AI integration in healthcare (22). American healthcare stakeholders have shown higher acceptance of ChatGPT in medical research compared to its use in healthcare and education (23), while healthcare workers in Saudi Arabia have recognized its utility but expressed concerns about its accuracy and reliability (24). An assessment of ChatGPT’s feasibility in patient-provider communication in the United States discovered moderate trust levels, which decreased with the complexity of the questions (25). Additionally, an experimental study in the UK found that while most participants preferred doctors for consultations, chatbots were favored for handling embarrassing symptoms, suggesting a nuanced role for AI in patient interactions (26).

These findings collectively highlight the complex landscape of generative AI acceptance, marked by differing perceptions across user demographics and professional backgrounds. They underscore the need for tailored approaches to effectively integrate ChatGPT into various settings, particularly in healthcare, where developing trustworthy AI tools that complement traditional services is crucial.

While frameworks like the Technology Acceptance Model (TAM) and Uses and Gratifications Theory have significantly advanced our understanding of user acceptance and engagement with AI technologies such as ChatGPT, challenges persist in effectively applying these insights to healthcare settings. TAM, originating from Davis, focuses on understanding how users accept and utilize new technologies, with core concepts including perceived ease of use and perceived usefulness (27). This model has been widely applied in many technology adoption studies, such as those involving ChatGPT (16, 17, 20), including in healthcare (15). On the other hand, Uses and Gratifications Theory explores the motivations and gratifications of individuals when using new technologies. Initially proposed by Katz et al. (28), this theoretical framework has been extensively used in media and technology studies, including those on ChatGPT (18, 19). These theoretical frameworks provide valuable perspectives for analyzing user behavior, trust levels, and risk perceptions regarding ChatGPT. However, existing research predominantly focuses on general user populations or specific educational contexts, often overlooking the nuanced needs and perceptions of healthcare professionals and patients. Additionally, the varying factors influencing AI adoption across different demographics and cultural backgrounds remain underexplored, particularly in healthcare, where the stakes are higher due to the direct impact on patient outcomes.

This study aims to bridge these gaps by investigating how general perceptions of ChatGPT as a generative AI influence its adoption and integration into healthcare, with a particular focus on differences between individuals with and without healthcare-related education. By examining attributes such as ease of use, novelty, perceived usefulness, trust, and risk, derived from both empirical studies and theoretical frameworks like TAM and Uses and Gratifications Theory, we seek to provide a comprehensive understanding of the factors driving AI acceptance in healthcare. This investigation will contribute to the development of more effective and tailored AI solutions that meet the unique needs of this critical sector.

Table 1 provides a summary of key factors from studies that explored ChatGPT’s general and healthcare-specific applications. It highlights how ease of use, novelty, and perceived usefulness significantly influence general adoption, while usefulness, trust and risk perceptions play a critical role in healthcare-specific applications. While general studies often rely on existing or modified question sets, the domain-specific question sets used in healthcare contexts were compiled in this study, drawing from a variety of review and survey research.

**Table 1:** Key factors in studies investigating ChatGPT’s general and healthcare-specific applications.

Factor	ChatGPT’s application for general purpose	ChatGPT’s domain-specific application in healthcare
Ease of use	*Populations studied:* General user populations, including students from Oman and Middle Eastern healthcare students. *Summary:* Ease of use was found to influence general acceptance, particularly in educational settings, though it was not always a significant factor for adoption (15–17, 20).	
Novelty	*Populations studied:* General user populations, including a Chinese study focused on users exploring new AI tools. *Summary:* Novelty was a significant predictor for performance expectancy (usefulness), which subsequently influenced the adoption of ChatGP (20).	
Usefulness	*Populations studied:* General user populations, including university students and professionals. *Summary:* Perceived usefulness was a major factor driving the adoption of ChatGPT for various tasks, from productivity enhancement to educational support (15–17, 20, 29).	*Populations studied:* Healthcare professionals, students, and patients across regions like the U.S., Europe, Asia, and Middle East. *Healthcare applications:* ChatGPT’s usefulness in healthcare was typically categorized into clinical practice, medical education, and medical research (1, 23), and widely acknowledged for enhancing workflow, patient support, teaching, learning, and research advancement, thereby contributing to improved decision-making and efficiency in healthcare settings (2, 3, 11–13, 24).
Trust	*Populations studied:* General user populations, including American users who value AI’s personalization capabilities. *Summary:* Trust in ChatGPT was enhanced by personalization and the perceived reliability of its responses, leading to higher user engagement and satisfaction (19, 21).	*Populations studied:* Healthcare professionals, patients, and general public from the U.S. and Europe. *Healthcare applications*: Trust was critical for the acceptance of ChatGPT in clinical settings, particularly in diagnostic support and patient communication, where accuracy and reliability are paramount (2, 5, 7, 10, 23, 25, 26).
Risk	*Populations studied:* General user populations, including those who have expressed concerns about AI’s potential risks. *Summary:* Perceived risks, such as privacy concerns and misinformation, affected user acceptance, with some users preferring more controlled environments (15, 16, 29, 30).	*Populations studied:* Healthcare workers, patients, and stakeholders in regions including the U.S., Europe, Middle East and China. *Healthcare applications:* The perception of risk, particularly concerning accuracy, liability, and data security, was a significant barrier to ChatGPT’s integration into healthcare practices, underscoring the need for rigorous safeguards (1–4, 6, 8, 9, 12, 22, 24).

In conclusion, comprehensive research on ChatGPT has underscored its strengths and limitations across clinical, educational, and research contexts, focusing on crucial issues like accuracy, transparency, and ethical standards. Theoretical frameworks such as TAM and Uses and Gratifications Theory offer valuable perspectives for analyzing user behavior, trust levels, and risk perceptions. However, a gap remains in understanding how general user perceptions translate into practical healthcare applications, emphasizing the need for further research.

Building on this foundation, our primary research objective is to explore the relationship between general perceptions of ChatGPT and its specific applications in healthcare, particularly in clinical practice, medical education, and research. We aim to assess how attributes such as perceived usefulness, trust, risk, ease of use, and novelty impact the perception of ChatGPT’s implementation in these areas. Additionally, this study will examine how these perceptions differ between individuals with and without healthcare-related education, providing insights into how educational background influences technology adoption in healthcare contexts.

Main research question:

How do general perceptions of ChatGPT (ease of use, novelty, perceived usefulness, trust, and risk) influence its perceived usefulness, trust, and risk in specific healthcare applications among different user groups (healthcare majors and non-healthcare majors)?

Sub-questions:

How do these perceptions influence the perceived usefulness, trust, and risk of ChatGPT in healthcare applications among healthcare majors?

How do these perceptions influence the perceived usefulness, trust, and risk of ChatGPT in healthcare applications among non-healthcare majors?

## Materials and methods

### Participants

Participants for this study were recruited through an online survey, with eligibility limited to those who had prior experience using ChatGPT. Recruitment was conducted using a convenience-based approach, targeting current students through email invitations distributed via university mailing lists, social media platforms, and online forums focused on healthcare and technology. While convenience sampling was chosen for its practicality and cost-effectiveness, we acknowledge its potential biases and limited generalizability. To mitigate these biases, efforts were made to recruit a diverse group of students across various disciplines and demographics, and the sample size was sufficiently large to enhance the robustness of the findings. The survey, hosted on a secure platform, was accessible for 4 weeks in March 2024. Participants were informed about the study’s objectives and the voluntary nature of their participation at the beginning of the survey, and they were required to provide informed consent electronically before proceeding. To ensure high data quality and reduce dropout rates, follow-up reminders were sent, and the survey was designed to be completed in approximately 20 min.

The inclusion criteria for this study encompassed current students who had experience using ChatGPT. The exclusion criteria included records from students in fields with smaller sample sizes, such as art, law, and agriculture, as well as those from individuals who were unwilling to disclose their gender or who had already graduated. An initial sample of 808 records was collected, and based on these criteria, 149 records were excluded. The final sample comprised 659 university and graduate students from Taiwan, with an average age of 22.72 years (SD = 2.50). This included 488 university students and 171 graduate students. Among the participants, 266 were enrolled in healthcare-related majors and 393 in non-healthcare fields, with 455 identifying as female and 204 as male. This study was approved by the Ethics Committee of National Tsing Hua University (approval number: 11212HT159).

### Instrument

The survey consists of three parts: background information; ChatGPT usage frequency and contexts (such as information gathering, problem-solving, idea generation, and health-related queries); perceptions of ChatGPT’s applications for general purposes; and perceptions of ChatGPT’s domain-specific applications in healthcare. While the question sets for general purpose perceptions primarily utilize existing or modified instruments, the domain-specific question sets were specifically developed for this study. These were based on a diverse range of review and survey research conducted by the research team, which includes a professor from the Graduate Institute of Learning Sciences and Technologies as the first author and her PhD student as the second author. The team, with multiple years of experience executing projects funded by the Taiwan National Science and Technology Council in the field of medical education, refined the instrument following a small-scale pilot study. This pilot study involved discussions with students from healthcare backgrounds to revise the survey topics and a trial run of the survey to ensure its effectiveness in Taiwan’s context. SH-C first-year PhD student in Health and Human Performance at the University of Florida, contributed to the conceptualization of this manuscript and the analysis of the research findings’ implications. In this study, some questionnaire items were specifically developed based on concepts from the literature, while others were adapted from existing instruments. Exploratory factor analysis (EFA) and reliability analysis confirmed the validity and reliability of the questionnaire, identifying distinct factors related to perceptions of ChatGPT’s general-purpose and healthcare-specific applications, thus demonstrating the instrument’s suitability for further research. The complete instrument is shown in Table 2.

**Table 2:** Survey questions.

Construct		Item	Scale options and scores
Frequency of using ChatGPT		I used ChatGPT	Almost every day: 5, more than once per week: 4, once per week: 3, at least once a month: 2, less than once a month: 1.
Contexts for using ChatGPT		Information gathering	5-point frequency Likert scale (always: 5, often: 4, sometimes: 3, seldom: 2, never: 1)
		Problem-solving	
		Generating ideas	
		Health-related queries	
Perceptions toward using ChatGPT for general purpose	Ease of use of ChatGPT (cronbach's α =0.88)	Learning how to use ChatGPT is easy for me.	5-point agreement Likert scale (strongly agree: 5, agree: 4, neutral: 3, disagree: 2, strongly disagree: 1)
		My interaction with ChatGPT is clear and understandable.	
		I found using ChatGPT is uncomplicated.	
		It is easy for me to become skillful using ChatGPT.	
	Novelty of ChatGPT (cronbach's α = 0.85)	I find using ChatGPT to be a novel experience.	5-point agreement Likert scale (strongly agree: 5, agree: 4, neutral: 3, disagree: 2, strongly disagree: 1)
		Using ChatGPT is new and refreshing.	
		Using ChatGPT satisfied my curiosity.	
		ChatGPT made me feel like I was exploring a new world.	
	Perceived usefulness of ChatGPT for general purpose (cronbach's α = 0.87)	I find using ChatGPT useful in daily life or work.	5-point agreement Likert scale (strongly agree: 5, agree: 4, neutral: 3, disagree: 2, strongly disagree: 1)
		Using ChatGPT would help me accomplish things more quickly.	
		Using ChatGPT has increased my productivity.	
		ChatGPT would increase my chances of achieving things that are important to me.	
	Trust of ChatGPT for general purpose (cronbach's α = 0.84)	ChatGPT is competent in providing the information and guidance I need	5-point agreement Likert scale (strongly agree: 5, agree: 4, neutral: 3, disagree: 2, strongly disagree: 1)
		ChatGPT is reliable in providing consistent and dependable information	
		ChatGPT is trustworthy in the sense that it is dependable and credible	
		ChatGPT is secure and protects my privacy and confidential information	
		ChatGPT is more useful than other sources of information that I have previously used.	
	Risk of ChatGPT for general purpose (cronbach's α = 0.81)	I am concerned about the reliability of the information provided.	5-point agreement Likert scale (strongly agree: 5, agree: 4, neutral: 3, disagree: 2, strongly disagree: 1)
		I am concerned about not knowing sources of information.	
		I am afraid that the use of ChatGPT would be a violation of academic and university policies.	
		I am afraid of relying too much on ChatGPT and not developing my critical thinking skills.	
		I am afraid of becoming too dependent on technology like ChatGPT.	
		I am afraid that using ChatGPT would result in a lack of originality.	
Domain-specific application in healthcare	Usefulness in clinical practice (cronbach's α = 0.67)	Using ChatGPT can improve the clinical workflow in the medical system (e.g., suggestions for the diagnosis and treatment procedure).	5-point agreement Likert scale (strongly agree: 5, agree: 4, neutral: 3, disagree: 2, strongly disagree: 1)
		Using ChatGPT can provide support to patients and their families in medical system (e.g., health information Q&A).	
	Usefulness in medical education (cronbach's α = 0.74)	Using ChatGPT can assist in enhancing the teaching effectiveness in medical education (e.g., generating course-related materials).	5-point agreement Likert scale (Strongly agree: 5, agree: 4, neutral: 3, disagree: 2, strongly disagree: 1)
		Using ChatGPT can assist in enhancing the learning outcomes in medical education (e.g., supporting student for personalized learning and self-directed learning).	
	Usefulness in medical research (cronbach's α = 0.76)	Using ChatGPT can support the advancement of academic progress in medical research (e.g., assisting with literature interpretation and scholarly writing).	5-point agreement Likert scale (strongly agree: 5, agree: 4, neutral: 3, disagree: 2, strongly disagree: 1)
		Using ChatGPT can support the advancement of academic community in medical research (e.g., assisting with knowledge dissemination and the framework of knowledge creation).	
	Trust (cronbach's α = 0.87)	I could trust answers from ChatGPT about logistical questions (such as scheduling appointments).	5-point agreement Likert scale (strongly agree: 5, agree: 4, neutral: 3, disagree: 2, strongly disagree: 1)
		I could trust ChatGPT to provide advice about preventative care.	
		I could trust ChatGPT to provide diagnostic advice about symptoms.	
		I could trust ChatGPT to provide treatment advice.	
		ChatGPT can be a more trustworthy alternative to Google to answer my health questions.	
		ChatGPT could help me make better health decisions.	
		I can imagine CharGPT or other Generative AI acting as a virtual doctor in the future.	
		We may encourage students from medical and healthcare to learn more about the development of ChaGPT and other Generative AIs.	
	Risk (cronbach's α = 0.89)	I am concerned about the ethical issue of using ChatGPT in medical domain (such as data bias and patience privacy).	5-point agreement Likert scale (strongly agree: 5, agree: 4, neutral: 3, disagree: 2, strongly disagree: 1)
		I am concerned about the issue of information accuracy when using ChatGPT in the medical domain.	
		I am concerned about the issue of information uninterpretability when using ChatGPT in the medical domain.	
		I am concerned about the regulatory issues when using ChatGPT in the medical domain.	
		I am concerned about the issue of knowledge copyright when using ChatGPT in the medical domain.	
		I am concerned about the issue of misinformation dissemination when using ChatGPT in the medical domain.	

For perceptions of ChatGPT’s general-purpose applications, there are five subscales:

*Ease of use* was assessed with four items (Cronbach’s *α* = 0.88), exemplified by “Learning how to use ChatGPT is easy for me.” Ease of use was assessed with four items (Cronbach’s *α* = 0.88), exemplified by “Learning how to use ChatGPT is easy for me,” with items adapted from (17, 20).

*Novelty* was assessed with four items (Cronbach’s *α* = 0.85), including “I find using ChatGPT to be a novel experience,” with items adapted from (20).

*Perceived usefulness* was measured by four items (Cronbach’s *α* = 0.87), featuring “I find using ChatGPT useful in daily life or work,” with items adapted from (20).

*Trust* was evaluated with five items (Cronbach’s *α* = 0.84), including “ChatGPT is reliable in providing consistent and dependable information,” and “ChatGPT is more useful than other sources of information that I have previously used,” with items adapted from (21).

*Risk* was assessed with six items (Cronbach’s *α* = 0.81), featuring “I am afraid of relying too much on ChatGPT and not developing my critical thinking skills,” and “I am afraid that the use of ChatGPT would be a violation of academic and university policies,” with items developed based on concepts discussed in (15, 29, 30).

For perceptions of ChatGPT’s domain-specific applications in healthcare, there are three main themes: Perceived Usefulness, Trust, and Risk. These are further divided into five subscales, with Perceived Usefulness broken down into its applications in clinical practice, medical education, and medical research, following the tripartite division outlined in (1, 23).

*Perceived usefulness in clinical practice* was assessed by two items (Cronbach’s *α* = 0.67), including “Using ChatGPT can improve the clinical workflow in the medical system (e.g., suggestions for diagnosis and treatment procedures)” and “Using ChatGPT can provide support to patients and their families in the medical system (e.g., health information Q&A),” with items developed based on concepts discussed in (2, 11, 24).

*Perceived usefulness in medical education* was assessed by two items (Cronbach’s *α* = 0.74), including “Using ChatGPT can assist in enhancing teaching effectiveness in medical education (e.g., generating course-related materials)” and “Using ChatGPT can assist in enhancing learning outcomes in medical education (e.g., supporting personalized and self-directed learning),” with items developed based on concepts discussed in (1, 3, 12).

*Perceived usefulness in medical research* was assessed by two items (Cronbach’s *α* = 0.76), including “Using ChatGPT can support the advancement of academic progress in medical research (e.g., assisting with literature interpretation and scholarly writing)” and “Using ChatGPT can support the academic community in medical research (e.g., assisting with knowledge dissemination and knowledge creation),” with items developed based on concepts discussed in (1, 3, 13).

*Trust* was measured by eight items (Cronbach’s *α* = 0.87), including “I could trust ChatGPT to provide advice about preventative care,” “to provide diagnostic advice about symptoms,” “to provide treatment advice,” and “to help me make better health decisions.” These eight items were primarily derived from Nov et al. (25) survey. In Nov et al. (25) study, participants were asked about their trust in chatbots for patient-provider communication. They evaluated their trust in chatbots to provide different types of services, including logistical information, preventative care advice, diagnostic advice, and treatment advice. Additionally, participants compared their trust in AI chatbots for answering health questions to that of a Google search and assessed their overall trust in AI chatbots to assist them in making better health decisions.

*Risk* was measured by six items (Cronbach’s *α* = 0.89), including “I am concerned about the ethical issues of using ChatGPT in the medical domain (such as data bias and patient privacy),” and “the issue of information accuracy when using ChatGPT in the medical domain,” with items developed based on concepts discussed and findings informed by the non-survey research papers in (1, 3, 6).

### Statistical analysis

For the purpose of this study, mean, SD, *T*-tests, and ANOVA were performed, and multiple linear regression models were conducted using 5 subscales on perceptions of ChatGPT’s domain-specific applications in healthcare as dependent variables: Perceived usefulness in clinical practice, Perceived usefulness in medical education, Perceived usefulness in medical research, Trust in healthcare applications, and Risk in healthcare applications. The independent variables are the 5 subscales on perceptions of ChatGPT’s general-purpose applications: Ease of use, Novelty, Perceived usefulness, Trust, and Risk. The regressions were performed separately for healthcare majors and non-healthcare majors, resulting in a total of 10 regression models.

## Results

### Descriptive analysis

The final sample comprised 659 university and graduate students from Taiwan, including 266 enrolled in healthcare-related majors and 393 in non-healthcare fields. Specifically, healthcare majors included 13.8% from the Medical Department, 13.1% from the Nursing Department, 7.9% from various health-related departments, and 5.6% from the Pharmacy Department. Non-healthcare majors were distributed across a range of fields: 10.8% from the College of Engineering, 9.9% from the College of Management, 9.4% from the College of Education, 9.3% from the College of Humanities and Social Sciences, 7.3% from the College of Electrical Engineering and Computer Science, 7.1% from the College of Science and Life Sciences, and 5.9% from the College of Information Management and Communication.

To explore ChatGPT usage patterns, we examined both the frequency and contexts of its use. Healthcare majors reported an average usage frequency of 2.20 (SD = 1.17) on a scale where 5 indicated almost every day, 4 more than once per week, 3 once per week, 2 at least once a month, and 1 less than once a month. In terms of usage contexts, they reported average scores of 3.48 (SD = 1.05) for problem-solving, 3.42 (SD = 1.07) for information gathering, 3.13 (SD = 1.13) for generating ideas, and 2.35 (SD = 1.18) for health-related queries, using a 5-point frequency Likert scale where 5 indicated always, 4 often, 3 sometimes, 2 seldom, and 1 never. In contrast, non-healthcare majors had an average usage frequency of 2.80 (SD = 1.20) and reported scores of 3.70 (SD = 0.96) for problem-solving, 3.64 (SD = 0.99) for information gathering, 3.43 (SD = 1.07) for generating ideas, and 1.98 (SD = 1.00) for health-related queries. *T*-test results revealed that non-healthcare majors use ChatGPT significantly more often than healthcare majors [*t*_(657)_ = 6.40, *p* < 0.001], with significant group differences in all four contexts: non-healthcare majors use ChatGPT more frequently for problem-solving [*t*_(532.479)_ = 2.722, *p* = 0.007], information gathering [*t*_(657)_ = 2.747, *p* = 0.006], and idea generation [*t*_(657)_ = 3.426, *p* < 0.001], while healthcare majors use it significantly more for health-related queries [*t*_(503.317)_ = −4.162, *p* < 0.001]. These findings suggest that the field of study influences how students engage with AI tools like ChatGPT, potentially reflecting differing academic and professional needs.

For the research variables, healthcare majors reported average scores of 4.02 (SD = 0.74) for ease of use, 4.19 (SD = 0.64) for novelty, 4.11 (SD = 0.69) for usefulness, 3.51 (SD = 0.77) for trust, and 3.76 (SD = 0.77) for risk in their perceptions of using ChatGPT for general purposes, based on a 5-point agreement Likert scale where 5 indicated strongly agree, 4 agree, 3 neutral, 2 disagree, and 1 strongly disagree. In terms of ChatGPT’s domain-specific applications in healthcare, they reported average scores of 3.70 (SD = 0.81) for usefulness in clinical practice, 3.85 (SD = 0.78) for usefulness in medical education, 3.84 (SD = 0.80) for usefulness in medical research, 3.24 (SD = 0.73) for trust, and 3.84 (SD = 0.74) for risk. Conversely, non-healthcare majors reported average scores of 4.06 (SD = 0.66) for ease of use, 4.10 (SD = 0.65) for novelty, 4.17 (SD = 0.60) for usefulness, 3.51 (SD = 0.65) for trust, and 3.77 (SD = 0.67) for risk. They also reported scores of 3.79 (SD = 0.68) for usefulness in clinical practice, 3.93 (SD = 0.67) for usefulness in medical education, 3.89 (SD = 0.68) for usefulness in medical research, 3.33 (SD = 0.65) for trust, and 3.84 (SD = 0.64) for risk. Although non-healthcare majors had slightly higher average scores than healthcare majors across almost all items, these differences were not statistically significant: in ChatGPT’s general-purpose applications: for ease of use, *t*_(657)_ = 0.812, *p* = 0.417; for novelty, *t*_(519.553)_ = 1.216, *p* = 0.225; for usefulness, *t*_(657)_ = 1.246, *p* = 0.213; for risk, *t*_(657)_ = −0.164, *p* = 0.87; for trust, *t*_(501.654)_ = 0.011, *p* = 0.991. In domain-specific healthcare applications: for usefulness in clinical practice, *t*_(500.608)_ = 1.607, *p* = 0.109; for usefulness in medical education, *t*_(511.026)_ = 1.305, *p* = 0.192; for usefulness in medical research, *t*_(506.845)_ = 0.808, *p* = 0.419; for trust, *t*_(657)_ = 1.76, *p* = 0.079; for risk, *t*_(514.05)_ = −0.06, *p* = 0.952.

Additionally, a one-way repeated measures ANOVA was conducted to compare the perceived usefulness of ChatGPT in clinical practice, medical education, and medical research among healthcare majors. The results revealed significant differences [*F*_(1.933, 512.287)_ = 6.276, *p* = 0.002, $\eta_{p}^{2}$ = 0.023]. Then, *post-hoc* tests were performed using pairwise comparisons. Clinical practice receiving significantly lower scores compared to medical education (MD = −0.152, *p* = 0.002) and research (MD = -141, *p* = 0.007). However, there was no significant difference between the scores of medical education and research (MD = 0.011, *p* = 0.798). This pattern was similarly observed among non-healthcare majors [*F*_(1.912, 749.581)_ = 8.721, *p* < 0.001, $\eta_{p}^{2}$ = 0.022]. *Post-hoc* tests showed that clinical practice scored significantly lower than both medical education (MD = −0.132, *p* < 0.001) and research (MD = −0.093, *p* = 0.009), with no significant difference between the latter two (MD = 0.039, *p* = 0.183). These findings suggest that while ChatGPT is valued for its educational and research potential, its application in clinical practice may face greater scrutiny and require more rigorous validation to gain similar levels of acceptance.

In summary, both healthcare and non-healthcare majors showed positive perceptions of ChatGPT, although non-healthcare majors rated it slightly higher across all aspects. The application of ChatGPT in healthcare settings indicated particular strengths in education and research over clinical practice.

### Regressions

Furthermore, this study explores how healthcare majors’ and non-healthcare majors’ overall impressions of ChatGPT (such as ease of use, novelty, usefulness, trustworthiness, and perceived risks) influence its perceived utility in healthcare settings, including clinics, medical schools, and research environments. Regression model results indicate that for healthcare majors, general trust in ChatGPT emerges as the only significant predictor of its application in clinical practice, as detailed in Table 3. In the areas of medical education and medical research, both perceived general usefulness and trust in ChatGPT serve as significant predictors, highlighting the importance of trust and perceived usefulness for healthcare majors. Conversely, for non-healthcare majors, the primary significant predictor for the application of ChatGPT in clinical practice is the novelty of the technology, as indicated in Table 4. Meanwhile, in the domains of medical education and medical research, the novelty of ChatGPT, along with perceived general usefulness and trust, are significant predictors.

**Table 3:** Multiple linear regression for predicting usefulness in domain-specific healthcare applications of healthcare major students.

	Healthcare major								
	Domain-specific healthcare applications								
Predictors	Usefulness in clinical practice			Usefulness in medical education			Usefulness in medical research		
	B (SE)	*t*	*p*-value	B (SE)	t	*p*-value	B (SE)	*t*	*p*-value
(Constant)	2.121 (0.382)	5.551	<0.001	1.661 (0.362)	4.591	<0.001	2.118 (0.38)	5.577	<0.001
Ease of use	0.012 (0.081)	0.153	0.879	0.038 (0.077)	0.495	0.621	0.062 (0.081)	0.772	0.441
Novelty	0.071 (0.089)	0.790	0.430	0.106 (0.085)	1.258	0.210	0.054 (0.089)	0.611	0.542
Perceived usefulness for general purpose	0.094 (0.088)	1.068	0.286	0.231 (0.083)	2.773	0.006	0.208 (0.087)	2.383	0.018
Trust for general purpose	0.34 (0.081)	4.220	<0.001	0.235 (0.076)	3.085	0.002	0.221 (0.08)	2.765	0.006
Risk for general purpose	−0.092 (0.06)	−1.527	0.128	−0.048 (0.057)	−0.846	0.398	−0.103 (0.06)	−1.721	0.086
F	11.352			14.235			11.106		
DF	(5,260)			(5,260)			(5,260)		
*p*-value	<0.001			<0.001			<0.001		
Adjusted *R*^2^	0.163			0.200			0.160		

**Table 4:** Multiple linear regression for predicting usefulness in domain-specific healthcare applications of non-healthcare major students.

	Non-healthcare major								
	Domain-specific healthcare applications								
Predictors	Usefulness in clinical practice			Usefulness in medical education			Usefulness in medical research		
	B (SE)	*t*	*p*-value	B (SE)	t	*p*-value	B (SE)	*t*	*p*-value
(Constant)	2.139 (0.306)	7.000	<0.001	1.677 (0.296)	5.676	<0.001	2.026 (0.309)	6.565	<0.001
Ease of use	−0.082 (0.059)	−1.372	0.171	−0.047 (0.058)	−0.810	0.418	−0.098 (0.06)	−1.629	0.104
Novelty	0.316 (0.06)	5.309	<0.001	0.249 (0.058)	4.322	<0.001	0.142 (0.06)	2.365	0.019
Perceived usefulness for general purpose	0.094 (0.067)	1.405	0.161	0.179 (0.065)	2.750	0.006	0.237 (0.068)	3.490	0.001
Trust for general purpose	0.083 (0.061)	1.359	0.175	0.157 (0.059)	2.648	0.008	0.164 (0.062)	2.644	0.009
Risk for general purpose	−0.001 (0.048)	−0.017	0.987	0.03 (0.047)	0.654	0.514	0.029 (0.049)	0.588	0.557
F	12.027			17.152			11.632		
DF	(5,387)			(5,387)			(5,387)		
*p*-value	<0.001			<0.001			<0.001		
Adjusted *R*^2^	0.123			0.171			0.119		

On the other hand, healthcare majors’ overall impressions of ChatGPT substantially influence their trust and perceived risk when deploying the technology in specific healthcare settings, accounting for 29.5 and 35.3% of variance, respectively, as shown in Table 5. Notably, general trust in ChatGPT emerges as the sole significant predictor of their trust in its application within healthcare domains. In contrast, the assessment of risk associated with using ChatGPT in healthcare is determined by three principal factors: general trust, perceived general risks, and the ease of use of the technology. Specifically, a lower general trust and higher perceived general risks correlate with increased perceived risks in healthcare applications. Additionally, a notable observation is that greater ease of use is associated with higher perceived risks in healthcare-specific applications, suggesting that while ease of use generally promotes adoption, in healthcare, it may raise concerns about potential overreliance and the adequacy of critical oversight. This understanding of how ease of use influences perceived risk highlights the complex considerations healthcare professionals make when integrating new technologies into their practice.

**Table 5:** Multiple linear regression for predicting trust and risks in domain-specific healthcare applications of healthcare major students.

	Healthcare major					
	Domain-specific healthcare applications					
Predictors	Trust			Risk		
	B (SE)	*t*	*p*-value	B (SE)	*t*	*p*-value
(Constant)	1.544 (0.318)	4.851	<0.001	1.752 (0.306)	5.718	<0.001
Ease of use	−0.112 (0.068)	−1.658	0.098	0.131 (0.065)	2.015	0.045
Novelty	0.124 (0.074)	1.660	0.098	0.132 (0.072)	1.836	0.067
Perceived usefulness for general purpose	0.047 (0.073)	0.640	0.522	0.1 (0.07)	1.415	0.158
Trust for general purpose	0.495 (0.067)	7.388	<0.001	−0.335 (0.065)	−5.192	<0.001
Risk for general purpose	−0.079 (0.05)	−1.571	0.117	0.472 (0.048)	9.742	<0.001
F	23.134			29.854		
DF	(5,260)			(5,260)		
*p*-value	<0.001			<0.001		
Adjusted *R*^2^	0.295			0.353		

Transitioning to non-healthcare majors, the factors influencing their trust in using ChatGPT for specific applications in healthcare are rather different, as detailed in Table 6. Here, general trust, novelty, and ease of use significantly affect their trust, where an increase in general trust and perceived novelty, coupled with a decrease in ease of use, correlates with greater trust in ChatGPT’s application in healthcare. Paradoxically, while less ease of use increases trust, it also highlights a critical perspective: that a technology requiring more effort to use may be perceived as more robust or serious, enhancing trust among non-healthcare professionals. Conversely, their assessment of risk is heavily swayed by perceived general risks and diminished general trust, resulting in greater perceived risks in healthcare applications. This illustrates that non-healthcare majors, although drawn to the innovative aspects of technology, remain cautious, reflecting a complex approach to adopting AI tools in sensitive fields similar to their healthcare counterparts.

**Table 6:** Multiple linear regression for predicting trust and risks in domain-specific healthcare applications of non-healthcare major students.

	Non-healthcare major					
	Domain-specific healthcare applications					
Predictors	Trust			Risk		
	B (SE)	*t*	*p*-value	B (SE)	*t*	*p*-value
(Constant)	1.509 (0.266)	5.670	<0.001	2.498 (0.276)	9.064	<0.001
Ease of use of ChatGPT	−0.171 (0.052)	−3.301	0.001	0.097 (0.054)	1.801	0.072
Novelty of ChatGPT	0.153 (0.052)	2.939	0.003	0.077 (0.054)	1.428	0.154
Perceived usefulness for general purpose	0.104 (0.059)	1.771	0.077	0.077 (0.061)	1.268	0.206
Trust for general purpose	0.456 (0.054)	8.512	<0.001	−0.285 (0.055)	−5.150	<0.001
Risk for general purpose	−0.037 (0.042)	−0.881	0.379	0.347 (0.043)	7.990	<0.001
F	29.874			20.564		
DF	(5,387)			(5,387)		
*p*-value	<0.001			<0.001		
Adjusted *R*^2^	0.269			0.200		

## Discussion

### Main findings

Our study revealed that both healthcare and non-healthcare majors consider ChatGPT more useful in medical education and research than in clinical practice. Although there were no significant differences in perceived usefulness between education and research, healthcare majors rated its application in clinical practice lower, indicating concerns about the reliability and applicability of AI in direct patient care. Additionally, our study identified distinct patterns in how these two groups perceive and adopt ChatGPT in medical contexts. Healthcare majors emphasize trust as a crucial factor in adopting ChatGPT across clinical practice, education, and research, showing a strong preference for AI tools that are reliable and credible. In contrast, non-healthcare majors are more influenced by ChatGPT’s novelty, especially in clinical practice, viewing it as an innovative tool. They also value the general usefulness and trustworthiness of ChatGPT, particularly in educational and research settings. These findings highlight the differing priorities between the two groups: healthcare majors prioritize trust and reliability, while non-healthcare majors are attracted to innovation and novelty.

These differing perceptions have significant implications for the practical integration of AI in healthcare settings. The lower confidence healthcare majors place in ChatGPT for clinical practice suggests that the integration of AI tools like ChatGPT in patient care may face resistance unless these tools can demonstrably meet the high standards of accuracy and reliability required in clinical settings. This highlights the need for ongoing validation and improvement of AI technologies to build trust among healthcare professionals. On the other hand, the enthusiasm for innovation among non-healthcare majors suggests that AI adoption in less critical areas such as education and research may be more straightforward, with fewer barriers to acceptance. However, this enthusiasm must be tempered with a critical understanding of AI’s limitations to ensure that its application is both responsible and effective. The contrast in perceptions underscores the importance of tailoring AI implementation strategies to address the specific concerns and priorities of different user groups within the healthcare ecosystem.

### Contrasting with existing literature

Results from the descriptive statistics revealed that, while prior research showed American healthcare stakeholders are more open to using ChatGPT in medical research than in clinical practice or educational applications (23), our findings indicate that both healthcare and non-healthcare groups in Taiwan rate clinical practice lower than medical education and research, with no significant differences observed between the latter two categories.

Our findings align with previous research that emphasizes the importance of general trust in the adoption of AI technologies like ChatGPT (19, 21), especially in healthcare settings where patient outcomes and ethical considerations are paramount. For healthcare majors, trust in ChatGPT emerges as the primary determinant of its adoption, consistent with earlier studies that underscore the need for AI tools to demonstrate reliability and credibility in high-stakes environments (25). This reliance on trust suggests that healthcare professionals are cautious about adopting new technologies that could impact patient care, highlighting the critical need for AI tools that complement human judgment rather than replace it.

Conversely, the novelty of ChatGPT plays a more significant role for non-healthcare majors, who are attracted to its innovative features. This extends earlier findings on the importance of novelty in AI adoption (18, 20), suggesting that non-healthcare majors are more open to exploring new technologies, particularly when they perceive them as useful and trustworthy.

Interestingly, our study also reveals a paradox where non-healthcare majors associate increased complexity with greater trust, viewing more complex interfaces as indicators of thoroughness and reliability. This contrasts with healthcare majors, who view ease of use with caution, fearing that overly simplistic systems might undermine the rigorous analytical processes required in clinical practice. While extant literature has inconsistent findings on the role of ease of use on ChatGPT’s adoption (16, 17), our results suggest a subtle influence, where professional background significantly shapes perceptions in technology.

### Implications for clinical practice

In the domain of clinical practice, the findings underscore the necessity for AI tools like ChatGPT to build trust among healthcare professionals by demonstrating reliability and ethical integrity. The lower ratings for clinical applications suggest that future developments should focus on integrating AI in ways that complement human judgment, ensuring that the technology supports clinical rigor rather than undermines it. This approach could help alleviate concerns and foster greater acceptance of AI in clinical settings. By fostering a collaborative relationship between AI and healthcare professionals, where AI serves as a supportive tool rather than a substitute for human expertise, the healthcare sector can move toward a future where AI enhances the quality of care. This approach will not only increase the acceptance of AI in clinical settings but also ensure that these tools contribute meaningfully to patient outcomes and the overall integrity of medical practice.

### Implications for medical education

In the domain of medical education, the high ratings for ChatGPT’s usefulness suggest a strong acceptance of AI as a transformative tool capable of revolutionizing the training of healthcare professionals. This recognition highlights the potential of ChatGPT to offer personalized learning experiences, tailor educational content to individual needs, and enhance the overall process of knowledge acquisition. By integrating AI into educational frameworks, institutions can develop innovative teaching methodologies that not only improve educational outcomes but also prepare students to navigate the complexities of modern healthcare environments. However, AI should serve as a tool to augment the learning process, enabling students to explore new ideas and approaches while maintaining the rigor and depth of traditional education. This collaboration will not only improve the quality of training for healthcare professionals but also ensure that graduates are well-prepared to leverage AI in their future clinical and research endeavors.

### Implications for medical research

In the domain of medical research, the favorable perceptions of ChatGPT’s utility reflect a growing acceptance of AI as a critical tool for advancing research practices. This acceptance underscores ChatGPT’s potential to enhance various aspects of academic work, including medical writing, literature interpretation, and scholarly communication. By supporting the advancement of academic progress and fostering the development of the academic community, ChatGPT aligns with the needs of researchers who seek efficient and innovative solutions to complex problems. However, just as in clinical practice and medical education, the adoption of AI in research must be approached with caution. Challenges such as the risks of misinformation, content inconsistency, and potential overdependence on AI highlight the need for careful management and oversight. Ensuring that AI tools like ChatGPT are used responsibly will be essential to maximizing their benefits while safeguarding the integrity of medical research.

### Implications for public health: responsible and ethical use of AI in healthcare

The integration of AI tools like ChatGPT into healthcare presents significant opportunities for improving public health, but it also raises critical ethical and operational challenges. To harness the benefits of AI while mitigating risks, healthcare professionals must prioritize transparency and trust in AI operations, ensuring that AI tools complement, rather than replace, clinical judgment. Continuous oversight and regulation are essential to prevent misuse and overdependence on technology, with a focus on understanding AI’s limitations, particularly in the patient care. Addressing risks such as misinformation and content inconsistency is crucial, requiring robust validation processes and regular updates to keep AI tools accurate and reliable. Additionally, equity and accessibility must guide AI adoption in healthcare, ensuring that all patients benefit from these advancements regardless of socioeconomic status or location. Ultimately, responsible and ethical use of AI in healthcare can enhance public health outcomes while maintaining the integrity and trust that are fundamental to patient care.

## Conclusion

The findings of this study emphasize the critical importance of designing AI tools that meet the distinct needs of healthcare and non-healthcare majors. For healthcare professionals, trust in AI technologies is paramount, particularly in clinical settings where the stakes are high. AI developers should focus on creating systems that are not only reliable and accurate but also transparent. By integrating explainable AI (XAI) features into tools like ChatGPT, developers can enhance credibility by providing clear, interactive explanations of how decisions are made. This transparency, which includes clear decision-making processes, the ability to customize based on context, and the capacity for auditability, is crucial for building trust and ensuring that AI tools are viewed as valuable supports for clinical judgment, rather than as replacements.

For non-healthcare majors, the appeal of innovation and novelty in AI tools suggests that developers should also focus on user-friendly interfaces and the integration of cutting-edge features that encourage exploration and engagement, especially in educational and research contexts. However, it is crucial that this enthusiasm is balanced with a clear understanding of AI’s limitations to prevent over-reliance or misuse.

### Future research directions

Future research should investigate the long-term impacts of AI adoption in healthcare, focusing on how sustained use affects clinical outcomes, patient trust, and the educational development of healthcare professionals. Qualitative studies, such as interviews and focus groups, are needed to explore the nuanced ways in which trust in AI is built and maintained across different user groups. Additionally, research should address the ethical implications of AI in healthcare, including issues related to patient privacy, data security, and the equitable distribution of AI benefits across different populations. Understanding these factors will be key to developing AI tools that are not only effective but also ethically sound and widely accepted.

### Limitations and contributions

This study acknowledges several limitations that could impact the generalizability of its findings. The use of convenience sampling and reliance on self-reported data may introduce biases and limit the depth of understanding of participants’ perceptions. Additionally, the focus on a specific demographic—students from Taiwan—means that the findings may not be fully applicable to other cultural or healthcare contexts. These limitations suggest that caution should be exercised when interpreting the results, particularly regarding their applicability to different populations.

To address these limitations, future research should incorporate more diverse sampling methods, including participants from multiple countries with varying cultural, socioeconomic, and educational backgrounds. Incorporating qualitative approaches, such as interviews or focus groups, would also provide richer insights into how trust in AI is built and maintained, allowing for a more nuanced understanding of user perceptions and behaviors.

Despite these limitations, this study offers valuable insights into the differing perceptions of AI among healthcare and non-healthcare students. The findings provide essential guidance for the development of AI tools that cater to the specific needs and concerns of these distinct groups. By addressing both the practical and ethical challenges of AI integration, this research contributes to the ongoing conversation about the role of AI in transforming healthcare and education.

## Data Availability

The raw data supporting the conclusions of this article will be made available by the authors, without undue reservation.
